# Hydroxychloroquine attenuates autoimmune hepatitis by suppressing the interaction of GRK2 with PI3K in T lymphocytes

**DOI:** 10.3389/fphar.2022.972397

**Published:** 2022-09-15

**Authors:** Chao Jin, Bei-Bei Gao, Wen-Jing Zhou, Bao-Jing Zhao, Xing Fang, Chun-Lan Yang, Xiao-Hua Wang, Quan Xia, Ting-Ting Liu

**Affiliations:** ^1^ School of Pharmacy, the First Affiliated Hospital of Anhui Medical University, Anhui Medical University, Hefei, China; ^2^ The Grade 3 Pharmaceutical Chemistry Laboratory of State Administration of Traditional Chinese Medicine, Hefei, China; ^3^ Department of Pharmacy, The Second Hospital of Anhui Medical University, Hefei, China; ^4^ Department of Obstetrics and Gynecology, The First Affiliated Hospital of Anhui Medical University, Hefei, China; ^5^ Department of Pharmacy, The Second People’s Hospital of Hefei, Hefei Hospital Affiliated to Anhui Medical University, Hefei, China

**Keywords:** hydroxychloroquine, autoimmune hepatitis, regulatory T cells, glycolipid metabolism, G protein-coupled receptor kinase 2, PI3K-AKT axis

## Abstract

Hydroxychloroquine (HCQ) is derivative of the heterocyclic aromatic compound quinoline, which has been used for the treatment of autoimmune diseases. The central purpose of this study was to investigate therapeutic effects and inflammatory immunological molecular mechanism of HCQ in experimental autoimmune hepatitis (AIH). Treatment with HCQ ameliorated hepatic pathologic damage, inflammatory infiltration, while promoted regulatory T cell (T_reg_) and down-regulated CD8^+^T cell differentiation in AIH mice induced by S-100 antigen. *In vitro*, HCQ also suppressed pro-inflammatory cytokine (IFN-γ, TNF-α, and IL-12) secretion, promoted anti-inflammatory cytokine (TGF-β_1_) secretion. HCQ mainly impaired T cell lipid metabolism but not glycolysis to promote T_reg_ differentiation and function. Mechanistically, HCQ down-regulated GRK2 membrane translocation in T cells, inhibited GRK2-PI3K interaction to reduce the PI3K recruiting to the membrane, followed by suppressing the phosphorylation of PI3K-AKT-mTOR signal. Pretreating T cells with paroxetine, a GRK2 inhibitor, disturbed HCQ effect to T cells. HCQ also reversed the activation of the PI3K-AKT axis by 740 Y-P (PI3K agonist). Meanwhile, HCQ inhibited the PI3K-AKT-mTOR, JAK2-STAT3-SOCS3 and increased the AMPK signals in the liver and T cells of AIH mice. In conclusion, HCQ exhibited specific and potent therapeutic effects on AIH and attendant liver injury, which was attributed to HCQ acted on GRK2 translocation, inhibited metabolism-related PI3K-AKT and inflammation-related JAK2-STAT3 signal in T lymphocytes, thereby modulating lipid metabolism of T cell function to regulate T_reg_ differentiation and function.

## Introduction

Autoimmune hepatitis (AIH) is a severe inflammatory liver disease, characterized by lymphocytic infiltration and immune cells imbalance, which induce the destruction of liver parenchyma and the elevated levels of transaminase (Yang et al., 2018). The current standard therapy of AIH is glucocorticoid alone or combinating with azathioprine, which would usually produce severe adverse effects (Christen, 2019). It is necessary to develop specific and safer therapeutic agents. Hydroxychloroquine (HCQ) is derivative of the heterocyclic aromatic compound quinoline, which has been used as antimalarial agent for a long time. Currently, it is an excellent candidate for the treatment of autoimmune diseases, such as systemic lupus erythematosus (SLE) and rheumatoid arthritis (RA) (Martinez et al., 2020). It was reported that the liver injury of COVID-19 patients could be alleviated with the treatment of HCQ (Sima et al., 2021). Other study found that HCQ probably have inhibitory effects on immune cellular infiltration and activation underlying joint inflammation (Ben-Zvi et al., 2012; Rainsford et al., 2015).

Defective immunoregulation in AIH might result from T_regs_ number and function reducing (Zhu et al., 2021). It was reported that HCQ rebalances Th17/T_reg_-mediated immunity and ameliorates SLE (An et al., 2017). Whether HCQ play a therapeutic role by regulating T_reg_ function in AIH has been attracted. Cell glucolipid metabolism is one of the key factors affecting T_reg_ differentiation (Matias et al., 2021). PI3K-AKT-mTOR pathway is reported to involve in immune cells metabolism (Xiang et al., 2016). mTOR inhibition could induce AMP-activated protein kinase (AMPK) activity and lipid oxidation in T_regs_, which promoted T_reg_ differentiation (Pompura and Dominguez-Villar, 2018).

G protein-coupled receptor kinases 2 (GRK2) is a key participant to modulate phosphorylation-dependent G protein coupled receptors (GPCRs) desensitization, endocytosis, intracellular trafficking and re-sensitization as well as the subsequent intracellular signaling cascades. Recent data indicated that GRK2 could interact with non-GPCR substrates such as PI3K, AKT, MEK and so on (Ribas et al., 2007), which involved in inflammatory, cardiovascular disease, and tumor treatment (Cheng et al., 2021). The composition of the C-terminal domain of approximately 230 amino acids allows GRK2 to combine with PI3K, AKT, PIP2, G_βγ_ and so on (Han et al., 2016). In RA, inhibiting the expression of GRK2 in membrane and increasing its expression in cytoplasm improved the abnormal proliferation of fibroblast like synovial cells. In addition, inhibition of GRK2 expression in rat spleen T cells can regulate T cell function to alleviate RA (Wang et al., 2017a; Wang et al., 2018). In the fibroblast-like synoviocytes of RA patients, the interaction of GRK2 with PI3Kγ promoted PI3K to recruit to the membrane, which contributed to the signal transduction (Wang et al., 2020). Other study showed that GRK2 also play a crucial role in function and differentiation of T_regs_ due to the interaction of GRK2 with PI3K-AKT pathway (Han et al., 2020).

Based on these observations, we hypothesize that HCQ ameliorates AIH by suppressing the inflammatory T-cell activity and promoting T_reg_ differentiation. To explore this, we detected the role of HCQ in AIH therapy and mechanism *in vivo* and *in vitro*. The results suggested that HCQ attenuated inflammation by regulating T cells lipid metabolism and T_reg_ differentiation, which was attributed to promoting the interaction of GRK2 with PI3K in the cytoplasm of T lymphocyte and inhibiting PI3K-AKT axis.

## Materials and methods

### Experimental autoimmune hepatitis model and treatment

Six-week-old male C57BL/6 mice were provided by Laboratory animal center of Anhui Medical University. All animal experimental procedures were approved by the Laboratory Animal Ethics Committee of Anhui Medical University (No. LLSC20190534). Mice were randomly divided into six groups (*n* = 6 per group) including control, AIH model, AIH+HCQ 10 mg/kg, AIH+HCQ 20 mg/kg, AIH+Prednisone (PRE) 8 mg/kg (positive control), AIH+HCQ 20 mg/kg+ PRE 8 mg/kg (drug combination) and control+HCQ 20 mg/kg (biosafety of HCQ). Hepatic syngeneic liver antigen (S-100) preparation: under non-sterile culture conditions, the livers of four female C57BL/6 mice were removed and cut into pieces on the surface of ice, then grinded on glass slides. After PBS washing, the protein in the liver cells was fully released and supernatant fluid was collected by ultracentrifugation. The experimental group was administered by intraperitoneal injection S-100 after fully emulsified on 1st day and 7th day with 0.5 ml of 0.5–2.0 g/L and an equal volume of complete freund’s adjuvant (CFA) (Beyotime, China). On the 14th day of modeling, the drug was administered by gavage for 2 weeks until all mice were sacrificed. The control mice were orally administered with normal saline.

### Histopathology and immunohistochemistry staining

Liver tissues were fixed in 4% paraformaldehyde for more than 24 h. Then the fixed liver tissues were stained for hematoxylin and eosin (H&E). Sections were incubated overnight at 4°C with CD3 and F4/80 primary antibodies (Proteintech, China) and incubated with biotin-labeled secondary antibody for 30 min after washing with phosphate-buffered saline. Slices were stained with chromogen diaminobenzidine and hematoxylin, dehydrated, and then treated with xylene.

### ALT/AST/MDA/SOD assay

The levels of alanine aminotransferase (ALT), aspartate aminotransferase (AST), malondialdehyde (MDA) and superoxide dismutase (SOD) in the serum of mice were assessed using Kit (#C009-1-1, #C010-1-1, #A003-1 and #A001-1, Nanjing Jiancheng Bioengineering Institute, China) according to manufacturer’s instructions.

### Cell culture and drug treatment

Spleen T lymphocytes were collected from the spleen of mice and activated with concanavalin A (ConA, 0.1 μM) and incubated with HCQ using doses in our concentration screening tests (100, 50, and 25 μM) for 24 h. Bone marrow derived dendritic cells (BMDCs) were collected from the tibia and femur of AIH mice. DCs were cultured with cytokines GM-CSF (20 ng/ml) and IL-4 (10 ng/ml) (Liu et al., 2015). All cells were cultured in RPMI 1640 medium (#BC-M-017, Biochannel, China) with 10% fetal bovine serum (FBS; Biological Industries, Israel) and maintained at 37°C in an incubator with 5% CO_2_ (Liu et al., 2014; Fang et al., 2020).

### FCM analysis

Immune cells were analyzed by FCM. Briefly, the cells underwent staining with fluorochrome-antibodies (Multi Sciences Lianke Bio, China) targeting cell antigens at 4°C for 60 min. Tissue abrasive fluid and T cell suspension were stained with the FITC-CD4, PE-CD25, APC-Foxp3 to identify T_regs_ and APC-CD3, FITC-CD4, PE-CD8 to identify CD4^+^ or CD8^+^T cells. The expressions of Foxp3 were observed in CD4^+^CD25^+^ cell gate, and the expressions of CD4 and CD8 were observed in CD3^+^ cell gate. BMDCs were stained with FITC-CD11c, APC-CD86 and PE-MHC-II (Liu et al., 2015), the expressions of MHC-II and CD86 were calculated in CD11c^+^ cell gate. The gate was chosen by the compare between negative cells (without fluorescent dye) and single fluorescent dye or mixed fluorescent dye cells. Furthermore, mitochondrial membrane potential (MMP) of cells was evaluated using JC-1 molecular probes and analyzed by flow cytometry. In cells with a high MMP (ΔΨm >80–100 mV), JC-1 forms aggregates that emit red-orange fluorescence (wavelength, 590 nm), whereas in cells with low mitochondrial potential (ΔΨm <80–100 mV), JC-1 forms monomers that emit green fluorescence (wavelength, 525–530 nm). The high MMP exerted high ratio of JC-1 Red/JC-1 Green. T lymphocytes were stained with JC-1 and tested in FITC and PE channels. The gate was chosen by the clustering of cells.

### Membrane and cytoplasm protein extraction

Membrane and cytoplasm protein of T lymphocytes was extracted by membrane and cytosol protein extraction kit (#P0033, Beyotime, China). T lymphocytes were lysed with membrane protein extraction reagent A and centrifuged at 700 g for 10 min at 4°C. The supernatant was centrifuged at 14,000 g for 30 min at 4°C. The supernatant was cytoplasm protein and the precipitate was the membrane proteins, which was resuspended in membrane protein extraction reagent B.

### Western blot analysis

The total proteins of liver tissues and spleen T lymphocytes were extracted by RIPA (#P0013C, Beyotime, China) with phosphatase inhibitor and PMSF (#P0012S, Beyotime, China). Immunoblotting was performed as previously described (Xiang et al., 2022). Primary antibodies were listed in Supplementary Table S1. Secondary antibodies were as follows: HRP-conjugated affinipure goat anti-rabbit IgG (H+L) (#SA00001-2, Proteintech, China) and HRP-conjugated affinipure goat anti-rabbit IgG (H+L) (#SA00001-1, Proteintech, China). The protein samples were visualized using the ECL-chemiluminescent kit (#WBKLS0100, Millipore, United States) and analysed by ImageJ software.

### Quantitative real-time-PCR

Specifically, total RNA was extracted from liver tissues and spleen T lymphocytes by TRIzol reagent (#257401, Invitrogen, United States). RNA was reverse transcribed in to cDNA by reverse transcription system kit (#R222-01, Vazyme, China). The mRNA levels were measured by qPCR using SYBR Green qPCR Master Mix (#Q111-02/03, Vazyme, China) according to the manufacturer protocol. The primers were listed in Supplementary Table S2. Gene expression was normalized to expression of β-actin.

### Enzyme-linked immunosorbent assay, free fatty acid secretion and glucose uptake assay

Levels of transforming growth factor-β_1_ (TGF-β_1_), interferon-γ (IFN-γ), interleukin-12 (IL-12), and tumor necrosis factor-α (TNF-α) in cell culture supernatant and serum were assessed with the ELISA assay (Proteintech, China). Free fatty acid was examined with the free fatty acid assay kit (#A042-1-1, Nanjing Jiancheng Bioengineering Institute, China), glucose uptake was examined by the glucose oxidase-peroxidase method with test kit (#361510, Rongsheng, China).

### Cell counting Kit-8 assay

The proliferation of T cell was assessed using CCK-8 kit (#GK10001, GLPBIO, China). After stimulation, the reagent was added into the culture medium, and the mixture was maintained in an incubator comprising 5% CO_2_ + 95% air at 37°C for 2°h. The absorbance at 450 nm was detected utilizing a Microplate Reader.

### Co-immunoprecipitation

To examine the association between GRK2 and PI3K or AKT in spleen T lymphocytes under different conditions, total cell lysates were immunoprecipitated using anti-GRK2 antibody and analyzed by western blot with an anti-GRK2, anti-PI3K or anti-AKT. Briefly, the preparation of lysate is as described above for western blots, and then pre-cleared using IgG. The total protein (1,000 µg of each lysate sample) was incubated with GRK2 antibody (4 µl) on a rotating shaker at 4°C overnight, the Protein A/G was added with agarose and then the small ball (20 µl) was added to each tube and placed in the rotating shaker at 4°C for 2 h. Then the tube was washed three times with cracking buffer. Resin-bound immune complexes were boiled for 5 min after protein loading buffer was added. Finally, repeating steps as Western blot.

### Statistical analyses

All data were statistically analyzed using GraphPad Prism 8.0. The data were normally distributed and expressed by the mean ± standard deviation (SD). Multiple comparisons were carried out by one-way (ANOVA). Comparisons between two groups were performed using independent-sample t-tests. All experiments were performed at least three times, *p* < 0.05 was considered statistically significant.

## Results

### Hydroxychloroquine ameliorated hepatic pathologic damage and inflammatory infiltration of autoimmune hepatitis mice

**FIGURE 1:**
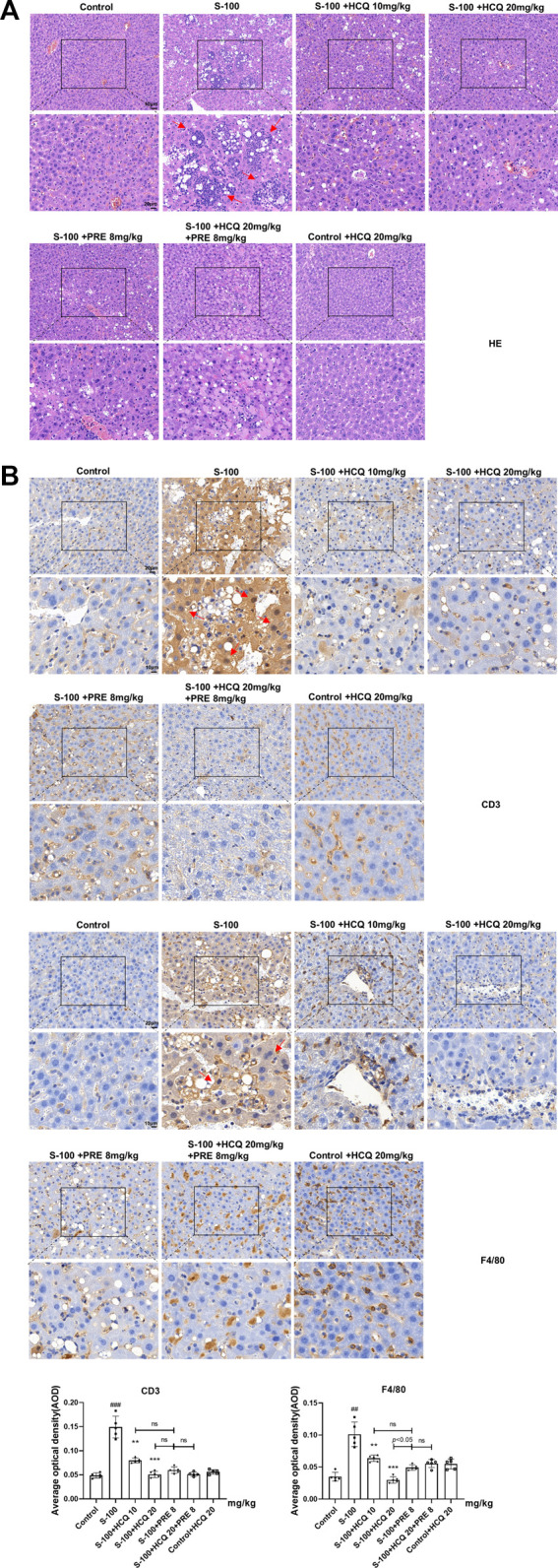
HCQ protected hepatic architecture and inhibited the infiltration of inflammatory cells in AIH mice. **(A)** Representative images of H&E (arrows represent inflammatory infiltrations, original magnification: ×200, scale bar of initial images: 50 μm; scale bar of enlarged images: 20 μm). **(B)** Representative immunohistochemical images of CD3 or F4/80 staining (arrows represent CD3 or F4/80 expression, original magnification: ×400, scale bar of initial images: 20 μm; scale bar of enlarged images: 10 μm). Data expressed as mean ± SD (*n* = 5). ^#^ *p* < 0.05, ^##^ *p* < 0.01, ^*###*^ *p* < 0.001 relative to controls; *p < 0.05, **p < 0.01, ***p < 0.001 relative to S-100-induced AIH mice; ns, not significant.

**FIGURE 2:**
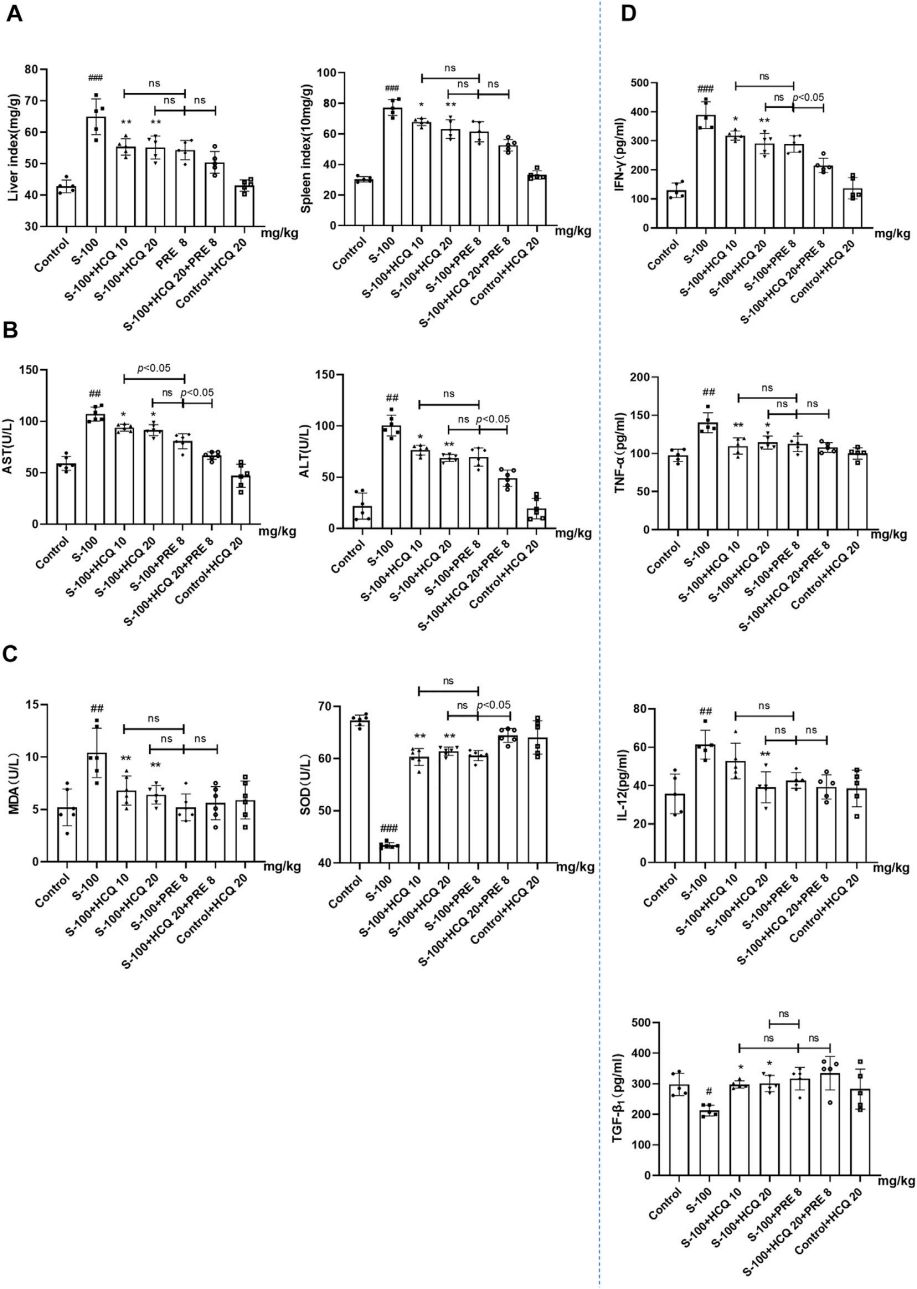
HCQ ameliorated hepatic pathologic damage and inflammatory infiltration of AIH mice. **(A)** Liver index [liver wet weight (mg)/mouse body weight (g) ×100%] and spleen index [spleen wet weight (10 mg)/mouse body weight (g) × 100%]. **(B)** Levels of ALT and AST in serum. **(C)** Levels of MDA and SOD in serum. **(D)** Serum inflammatory cytokine concentrations. Data expressed as mean ± SD (*n* = 5 or 6). ^#^ *p* < 0.05, ^##^ *p* < 0.01, ^*###*^ *p* < 0.001 relative to controls; *p < 0.05, **p < 0.01, ***p < 0.001 relative to S-100-induced AIH mice; ns, not significant.

In S-100-treated AIH mice, oral administration of 10 or 20 mg/kg HCQ, which referred to the report (Zheng et al., 2021), for 2°weeks markedly diminished the extent of AIH with the loss of liver architecture, congestion, lymphocytic infiltration and large area necrosis (arrows represent inflammatory infiltrations) (Figure 1A). We focused on inflammatory cell related markers, such as mature macrophages cell marker F4/80 and lymphocyte marker CD3 to investigate the alleviating effect of HCQ on the inflammatory infiltration of AIH. The results showed that the expression of CD3 and F4/80 significantly increased in the liver of AIH, which was slipped to basal levels by HCQ treatment (Figure 1B). The liver index and spleen index decreased in HCQ treatment compared with AIH group to a certain extent (Figure 2A). Serum levels of ALT and AST were decreased after HCQ treatment (Figure 2B). HCQ memorably attenuated hepatic oxidative stress with lower MDA and higher SOD compared with S-100 treatment (Figure 2C). Furthermore, the decrease of anti-inflammatory factor, TGF-β_1_ and the increase of proinflammatory factor, IFN-γ, TNF-α and IL-12 in serum caused by S-100 could be reversed by HCQ (Figure 2D). These effects were better or comparable to those of PRE, which was used in clinical settings against AIH. In addition, there was synergistic effect in treating AIH by combination of HCQ and PRE. Furthermore, HCQ treatment did not alter levels of ALT, AST, or inflammatory factors in healthy mice (Figures 1, 2). The results demonstrated that HCQ exerted liver-protective and anti-inflammatory effects on S-100-induced AIH and appeared to have no serious adverse effects.

### Hydroxychloroquine regulated T cells function and T_reg_ differentiation in autoimmune hepatitis

**FIGURE 3:**
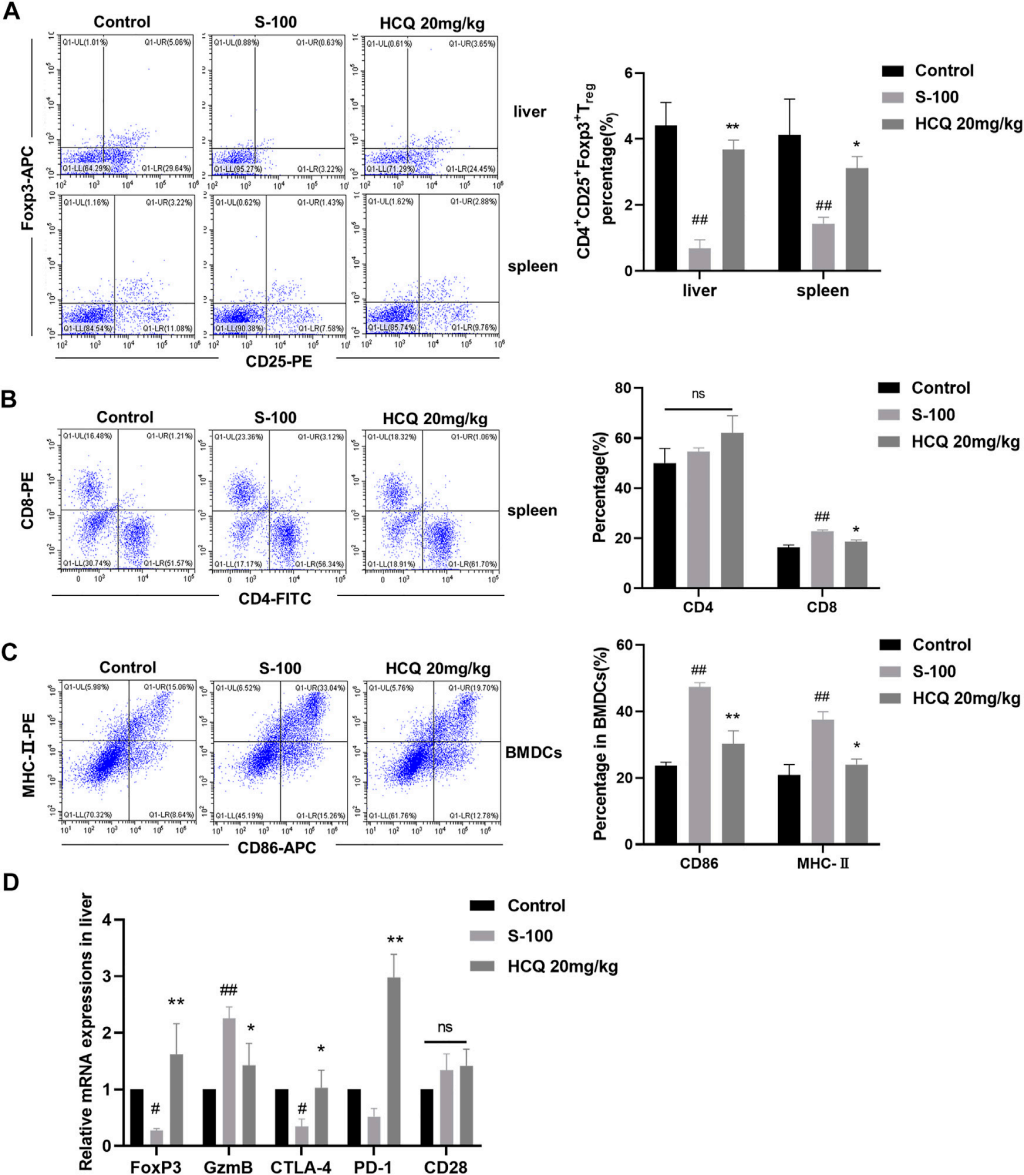
HCQ regulated immune cells differentiation in S-100-induced AIH. **(A)** The percentage of T_regs_ in the liver and spleen (*n* = 3). **(B)** The percentage of CD4^+^T cells and CD8^+^T cells in the liver (*n* = 3). **(C)** The MHC-II and CD86 expression on CD11c^+^ BMDCs (*n* = 3). **(D)** Expression of Foxp3, GzmB, CTLA-4, PD-1, CD28 mRNA in the liver (*n* = 5). Data expressed as mean ± SD. ^#^ *p* < 0.05, ^##^ *p* < 0.01 relative to controls; *p < 0.05, **p < 0.01 relative to S-100-induced AIH mice.

Numerous immune cells regulate the development of hepatic inflammation (Koyama and Brenner, 2017). We quantitatively determined the proportions of various immune cells of multiple organs in AIH. The results showed that, after S-100 administration, T_regs_ reduced in the spleen and liver, CD8^+^T cells differentiated in the spleen, and bone marrow derived dendritic cells (BMDCs) matured. HCQ reversed the effect of S-100 (Figures 3A–C), but had little effect on other subtypes of immune cells in the liver and spleen of AIH mice (Date not shown). Here, we also noted that HCQ increased the fork head box protein 3 (Foxp3) mRNA expressions in the liver of AIH mice, which facilitated T_regs_ immune tolerance. The serine protease granzyme B (GzmB), markers of CD8^+^T cell-induced cytotoxicity, was reduced by HCQ in the liver of AIH mice. HCQ showed a potential immunosuppressive effect on DC-CD8^+^T cell communication by upregulating the negative regulatory molecules CTLA-4 and PD-1 in the liver. The effect of HCQ to other costimulatory molecules were indistinctive (Figure 3D).

**FIGURE 4:**
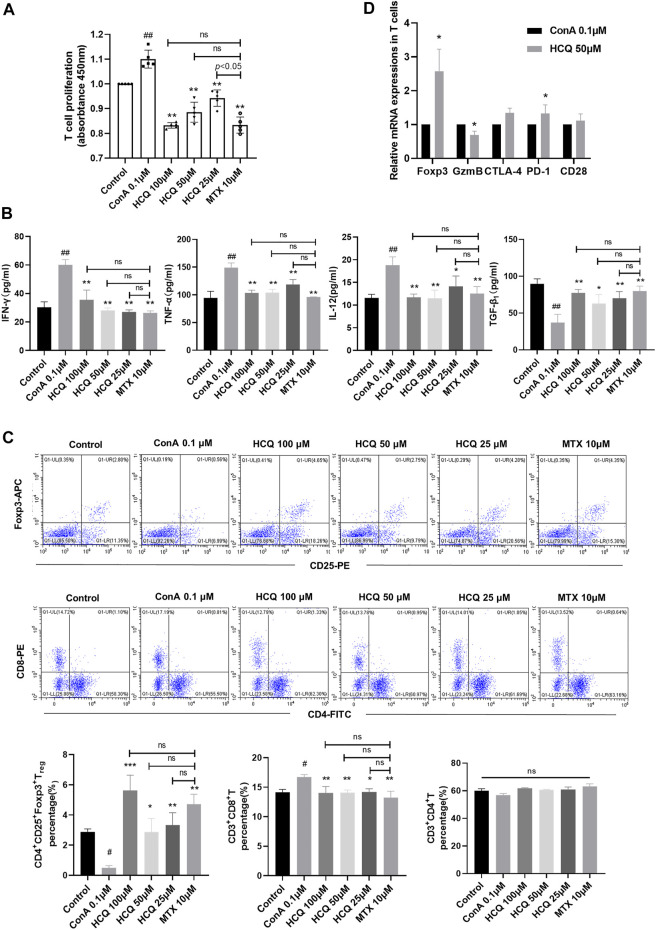
HCQ regulated T cell function and differentiation *in vitro*. **(A)** Proliferation of T cells (*n* = 5). **(B)** Inflammatory cytokine concentrations in T cell culture supernatant (*n* = 5). **(C)** The percentage of T_regs_, CD4^+^T cells, CD8^+^T cells in T lymphocytes (*n* = 3). **(D)** Expression of Foxp3, GzmB, CTLA-4, PD-1, CD28 mRNA in T lymphocytes (*n* = 5). Data expressed as mean ± SD. ^#^ *p* < 0.05, ^##^ *p* < 0.01 relative to controls; *p < 0.05, **p < 0.01, ***p < 0.001 relative to ConA group; ns, not significant.

*In vitro*, HCQ significantly suppressed spleen T-lymphocyte proliferation in a dose dependent manner (Figure 4A). It also decreased the pro-inflammatory cytokines, and increased the anti-inflammatory cytokines production in the supernatant of T cells (Figure 4B). These effects were comparable to those of MTX, which is used to inhibit T cells proliferation and function. Of note, *in vitro*, HCQ strongly increased T_regs_ and slightly retarded CD8^+^T cells differentiation (Figure 4C). HCQ represented similar effects on Foxp3 and GzmB mRNA expressions *in vitro* compared to *in vivo* experiment (Figure 4D). The apparent modulation involving T_regs_ of HCQ might be important to maintain immune homeostasis during progression of AIH.

### Impaired lipid metabolism in hydroxychloroquine-modulated T lymphocyte

**FIGURE 5:**
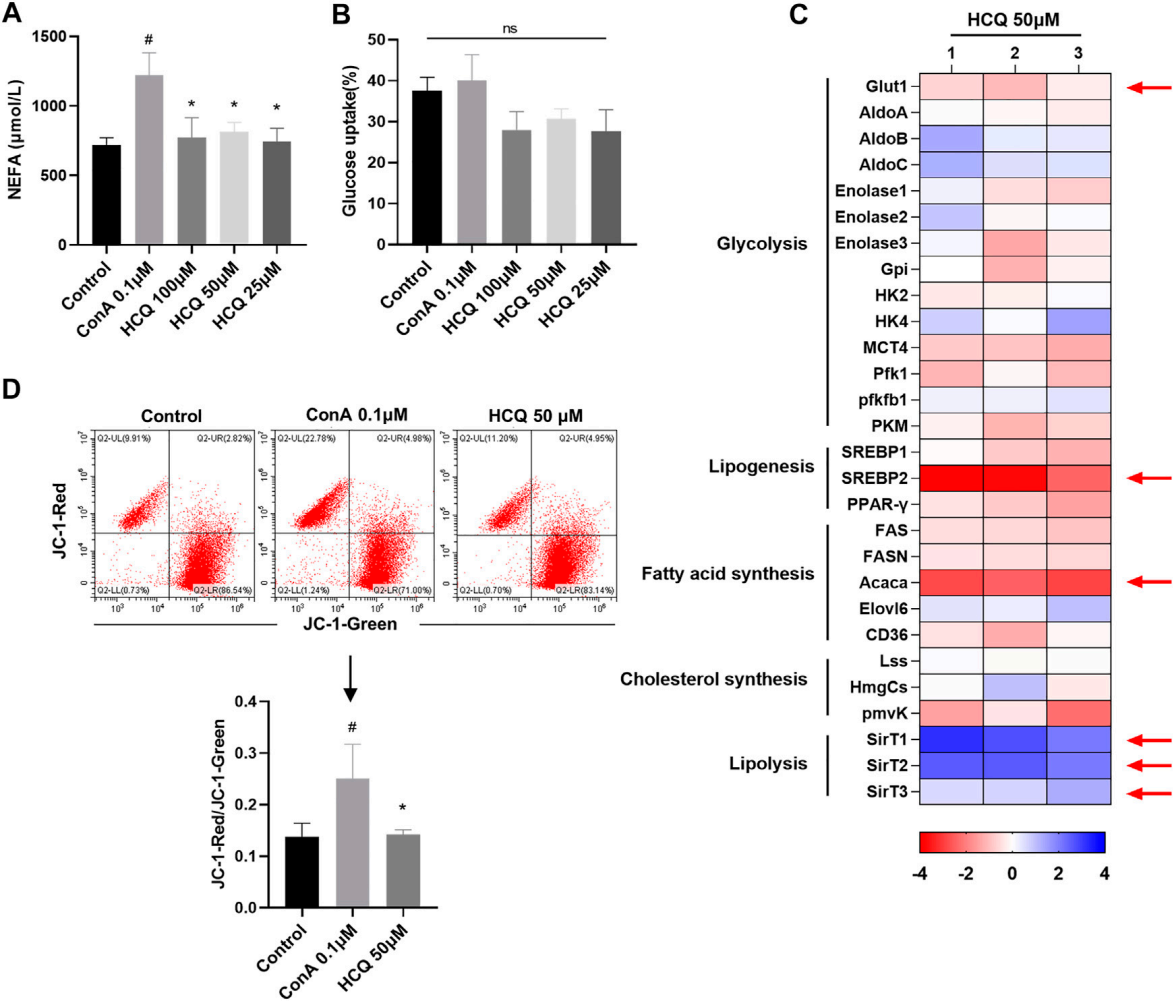
Lipid metabolism in T cells was controlled by HCQ. **(A)** Appearance of NEFA (*n* = 5). **(B)** Glucose uptake (*n* = 5). **(C)** Marker genes of glycolysis and lipid metabolism, mRNA levels in model controls were set to 1, the heat map represented the log_2_ value of the relative mRNA expression levels (see color scale), 1, 2, and 3 represent three parallel experiments. **(D)** MMP of T cells (*n* = 3). Data expressed as mean ± SD. ^#^ *p* < 0.05, ^##^ *p* < 0.01 relative to controls; *p < 0.05 relative to ConA.

Manipulating the metabolism of immune cells may alter immune homeostasis (Fox et al., 2005). Induced T_regs_ differentiate from conventional naïve CD4^+^T cells under a variety of conditions that range from inflammatory environments, in the presence of particular cytokines, mainly TGF-β_1_, to suboptimal glycolysis and/or fatty acid oxidation signals (Murphy et al., 1990). Previous reports indicated that HCQ exerted substantial metabolic regulation effects including lipid and insulin metabolism (Hu et al., 2017). Thus, HCQ might ameliorate AIH partly by suppressing T cells metabolism, which promoted T_reg_ development. Our results showed that HCQ inhibited NEFA secretion of T cells, but had little effect on T cell glucose uptake (Figures 5A,B). In the presence of HCQ, glucose transporter (GLUT)-1, the predominant glucose transporter in T cells, were lower compared to that of activated cells without treatment (Figure 5C). We found that HCQ treatment resulted in a significant reduction in MMP of T cells (Figure 5D). We also tested the marker genes of glycolysis and lipid metabolism [lipid synthesis and fatty acid oxidation (FAO)]. The result showed that there was no significant effect of HCQ to glycolysis in T cells, however, HCQ substantially downregulated mRNAs encoding lipid utilization components in T cells (*Srebp2* and *Acaca*), upregulated the fatty acid oxidation related genes expression (*SirT1*, *SirT2*, and *SirT3*), which might promote T_reg_ differentiation (Figure 5C). Given that mitochondria were central to the metabolism of lipid (Seenappa et al., 2016), we speculated that suppression of fuel metabolism and promotion of FAO might contribute importantly to the mechanism of T_reg_ development induced by HCQ.

### Hydroxychloroquine acted on G protein-coupled receptor kinases 2 translocation and reduced the membrane recruitment of PI3K in spleen T lymphocytes

The network pharmacology studies showed that multi-targets mechanism associated with HCQ treatment in SLE and RA. The related 3,316 proteins’ network data showed that ErbB, HIF-1, NF-κB, FoxO, chemokines, MAPK, JAK-STAT, PI3K-AKT pathways participate in the multi-targets mechanism of HCQ in RA (Lyu et al., 2020; Xie et al., 2021). The efficacy of HCQ against SLE is mainly associated with the targets of cyclin dependent kinase 2 (CDK2), estrogen receptor alpha (ESR1) and CDK1, which regulate PI3K/AKT/GSK3β as well as IFN signaling pathway (Xie et al., 2020). Another study showed that C-C chemokine receptor type 4 (CCR4), a GPCR modulated by GRK2, might an immunomodulatory target of HCQ (Beck et al., 2020). Our studies detected the protein expression of GRK2/PI3K-AKT signal in blood lymphocytes of RA patients and normal people, the results showed that the protein expression of GRK2, p-PI3K and p-AKT were increased in RA patients. The above indicators were reversed after treating by HCQ. In addition, the expression of GzmB was decreased, while the expression of Foxp3 was increased after HCQ treatment in the blood lymphocyte of RA patients (Data not shown). Based on above results and given that the GRK2/PI3K-AKT pathway contributes to the regulation of immune cell metabolism (Yang et al., 2020), we speculated that inhibiting the over activated PI3K-AKT pathway in immune cells might be effective methods for the treatment of RA and other autoimmune diseases such as AIH.

**FIGURE 6:**
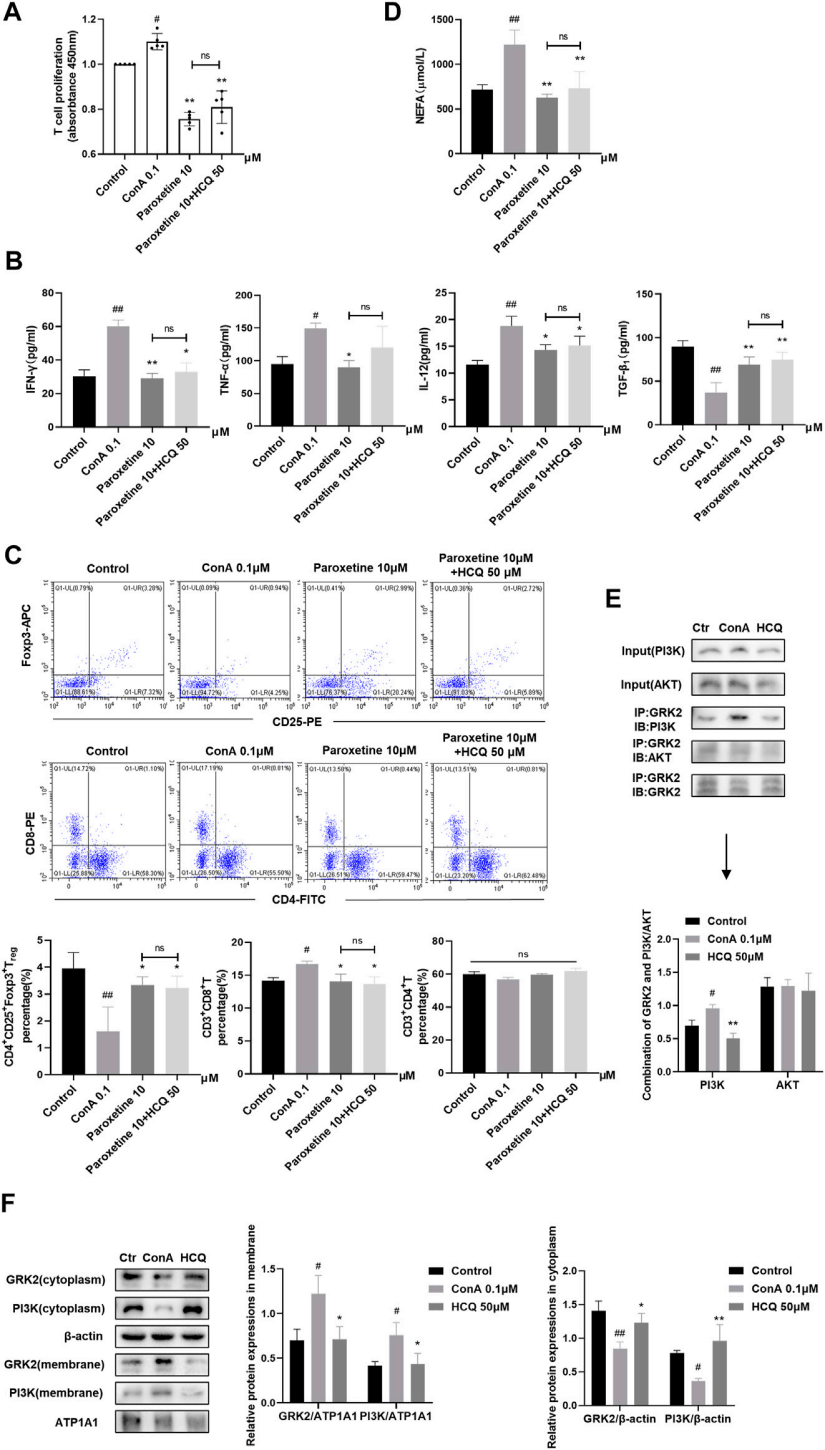
HCQ acted on GRK2 translocation and reduced the membrane recruitment of PI3K in spleen T lymphocytes **(A)** Proliferation of T cells after inhibiting GRK2 (*n* = 5). **(B)** Inflammatory cytokine concentrations in T cell culture supernatant after inhibiting GRK2 (*n* = 5). **(C)** The percentage of T_regs_, CD4^+^T cells, CD8^+^T cells in T lymphocytes after inhibiting GRK2 (*n* = 3). **(D)** Appearance of NEFA after inhibiting GRK2 (*n* = 5). **(E)** The combination of GRK2 and PI3K or AKT (*n* = 3). **(F)** Membrane and cytoplasm protein expression of GRK2 and PI3K (*n* = 3). Data expressed as mean ± SD. ^#^ *p* < 0.05, ^##^ *p* < 0.01 relative to controls; *p < 0.05, **p < 0.01 relative to ConA group; ns, not significant.

We pre-treated ConA induced T cells with paroxetine (GRK2 inhibitor), which retarded the proliferation, proinflammatory factor (IFN-γ, TNF-ɑ, and IL-12) secretion, and NEFA secretion, meanwhile, promoted T_reg_ differentiation and anti-inflammatory factor (TGF-β_1_) secretion. Adding HCQ did not enhance the effect of paroxetine, which suggested that the main target of HCQ might have been blocked (Figures 6A–D). Previous studies showed that the interaction of GRK2 with PI3Kγ promoted PI3K to recruit to the membrane to contribute to the signal transduction. Therefore, we detected the combination of GRK2 and PI3K by CO-IP and the membrane and cytoplasmic expression of GRK2 and PI3K by WB respectively after HCQ treatment. The results found that GRK2 and PI3K co-expression increased by ConA stimulation, and HCQ down-regulated GRK2 and PI3K interaction (Figure 6E). In addition, HCQ down-regulated GRK2 and PI3K translocation, inhibited the expression of GRK2 and PI3K in the cell membrane and increased their expression in the cytoplasm of spleen T lymphocytes (Figure 6F). Based on above results, we proposed that HCQ acted on GRK2, inhibited the interaction of GRK2-PI3K and their translocation, which reduced the recruitment of PI3K to the membrane, inhibited downstream signal transduction to disturb the function of activated T cells.

### Hydroxychloroquine decreased the activation of metabolism-related PI3K-AKT-mTOR and inflammation-related JAK2-STAT3-SOCS3 pathways in the T cells and liver tissue of AIH mice

**FIGURE 7:**
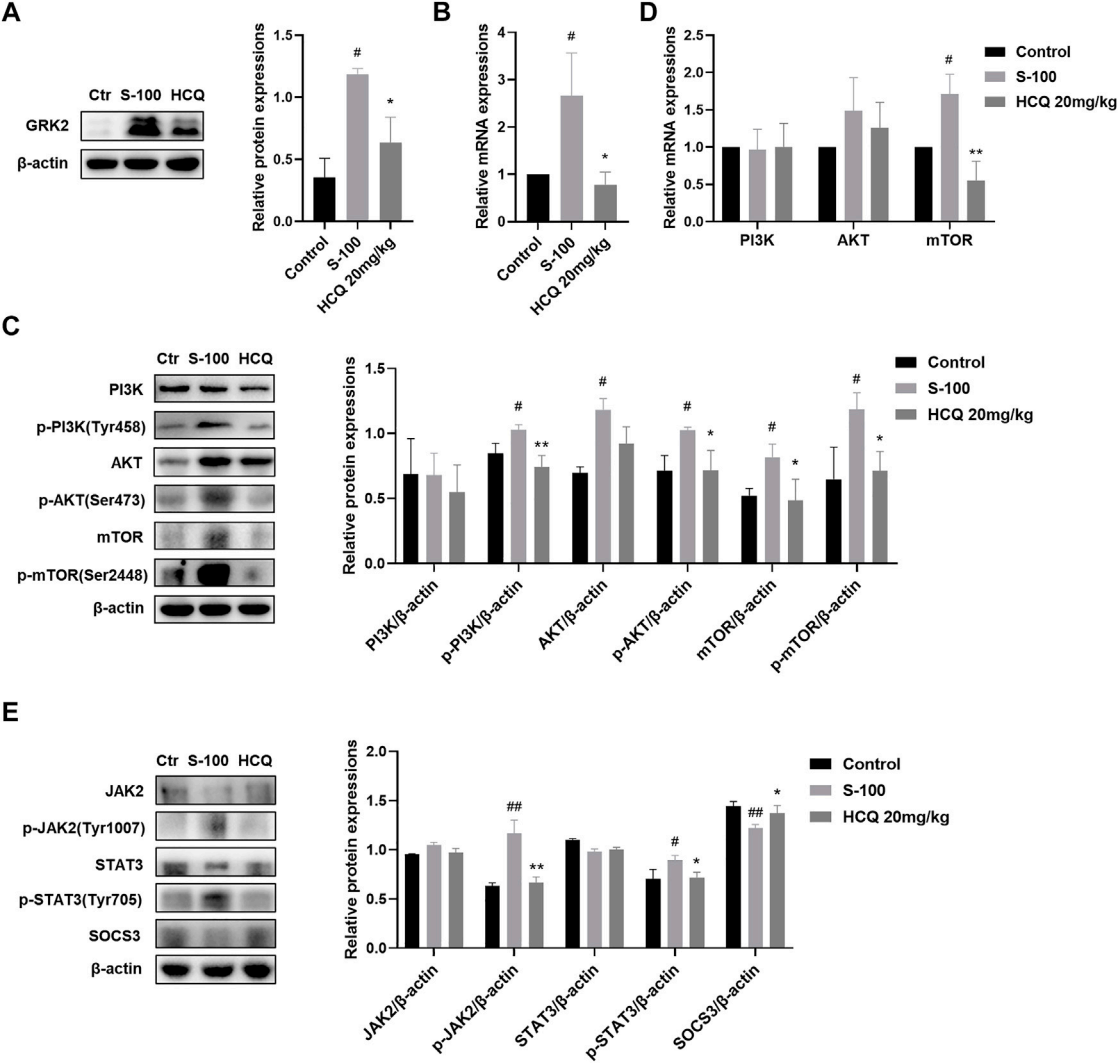
HCQ suppressed GRK2/PI3K-AKT-mTOR and JAK2-STAT3-SOCS3 pathways in the liver. **(A)** Total levels of GRK2 proteins in liver (*n* = 3). **(B)** Transcript levels of GRK2 in the liver (*n* = 5). **(C)** Total and phosphorylated levels of PI3K, AKT, and mTOR in the liver (*n* = 3). **(D)** Transcript levels of PI3K, AKT, and mTOR in the liver (*n* = 5). **(E)** Total and phosphorylated levels of JAK2, STAT3, and total levels of SOCS3 proteins in the liver (*n* = 3). Data expressed as mean ± SD. ^#^ *p* < 0.05, ^##^ *p* < 0.01 relative to controls; *p < 0.05, **p < 0.01 relative to S-100-induced AIH mice.

The PI3K-AKT pathway contributes to the regulation of immune cell metabolism (Yang et al., 2020), and JAK2-STAT3 pathway is widely considered to be involved in inflammation related diseases. In addition, it has been reported that HCQ may be involved in the regulation of these two pathways (Lyu et al., 2020; Li et al., 2022). Our results showed that HCQ treatment inhibited the protein and mRNA expression of GRK2 (Figures 7A,B) and reversed the activated PI3K-AKT-mTOR pathway induced by S-100 in the liver (Figure 7C). HCQ also suppressed phosphorylation of JAK2, STAT3, and increased SOCS3, the negative regulatory factor, in the liver of AIH mice (Figure 7D).

**FIGURE 8:**
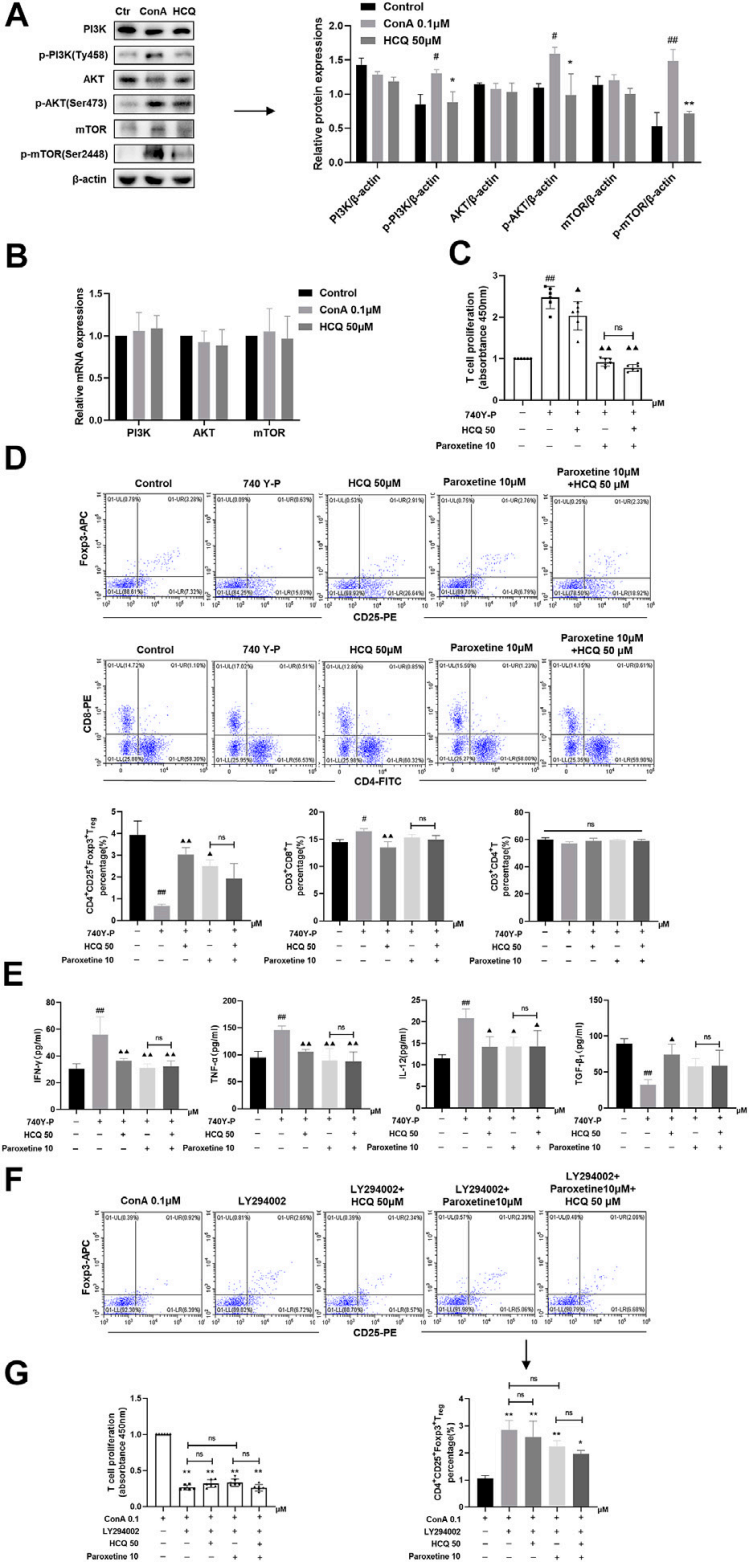
HCQ modulated T cell function and T_reg_ differentiation via the PI3K-AKT-mTOR pathway. **(A)** Total and phosphorylated levels of PI3K, AKT, and mTOR proteins of T lymphocytes (*n* = 3). **(B)** Transcript levels of PI3K, AKT, and mTOR in T lymphocytes (*n* = 5). **(C)** Proliferation of T cells (*n* = 6). **(D)** The percentage of T_regs_, CD4^+^T cells, CD8^+^T cells in T lymphocytes (*n* = 3). **(E)** Inflammatory cytokine concentrations in T cell culture supernatant (*n* = 5). T lymphocytes isolated from C57BL/6 J mice were incubated with 740 Y-P or 740 Y-P+HCQ. **(F)** The percentage of T_regs_ in T lymphocytes (*n* = 3). **(G)** Proliferation of T cells (*n* = 6). T lymphocytes isolated from C57BL/6 J mice were incubated with LY294002, LY294002+HCQ. Data expressed as mean ± SD. ^#^ *p* < 0.05, ^##^ *p* < 0.01 relative to controls; *p < 0.05, **p < 0.01 relative to ConA group; ^▲^ *p* < 0.05, ^▲▲^ *p* < 0.01 relative to 740Y-P; ns, not significant.

In T lymphocytes, HCQ suppressed phosphorylation of PI3K (Tyr458), AKT (Ser473) and mTOR (Ser2448), without affecting their mRNA and total protein expressions (Figures 8A,B). Following the PI3K agonist (740 Y-P, 20 μg/ml, 12 h)-mediated activation of PI3K, T cells exhibited proliferation, with enhanced levels of T_regs_, elevated secretion of IFN-γ, TNF-α and IL-12, and lowered expression TGF-β_1_. HCQ, on the other hand, eliminated the impact induced by 740Y-P (Figures 8C–E). In addition, PI3K inhibitors (LY294002, 10 μM, 12 h) inhibited T cell proliferation and promote T_reg_ differentiation compared with ConA group, adding HCQ did not enhance the effect of LY294002 (Figures 8F,G). GRK2 inhibitor also appeared to inhibit pathway activation in the present of 740 Y-P. And the effect of GRK2 inhibitor was weak after LY294002 treatment in T cells. The results suggested that HCQ effect on PI3K-AKT signal might be mediated by GRK2 (Figures 8C–G).

**FIGURE 9:**
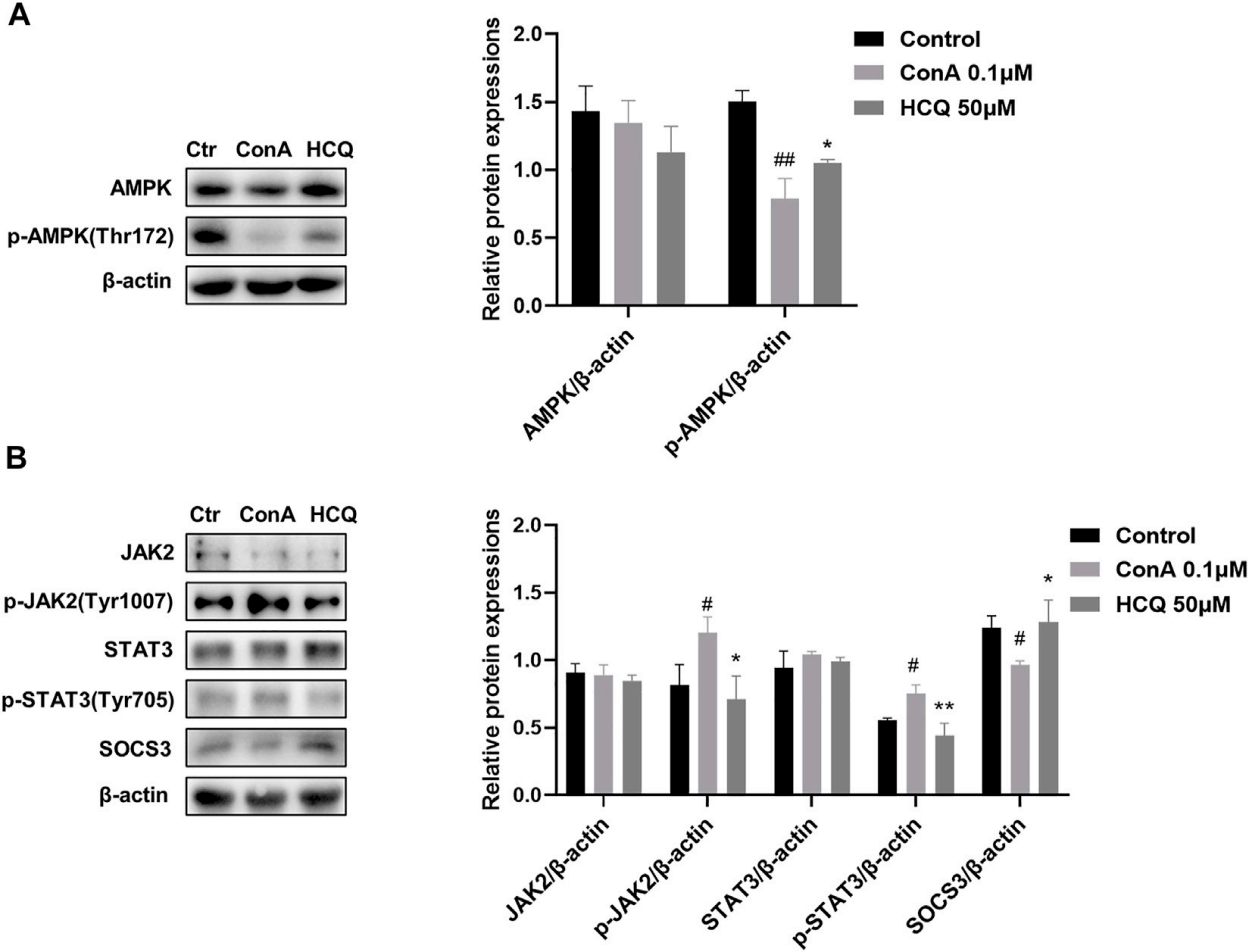
HCQ suppressed the activation of JAK2-STAT3-SOCS3 and promoted the phosphorylation of AMPK in T lymphocytes. **(A)** Total and phosphorylated levels of JAK2, STAT3, and total levels of SOCS3 proteins of T lymphocytes (*n* = 3). **(B)** Total and phosphorylated levels of AMPK of T lymphocytes (*n* = 3). Data expressed as mean ± SD. ^#^ *p* < 0.05, ^##^ *p* < 0.01 relative to controls; *p < 0.05, **p < 0.01 relative to ConA group.

**FIGURE 10:**
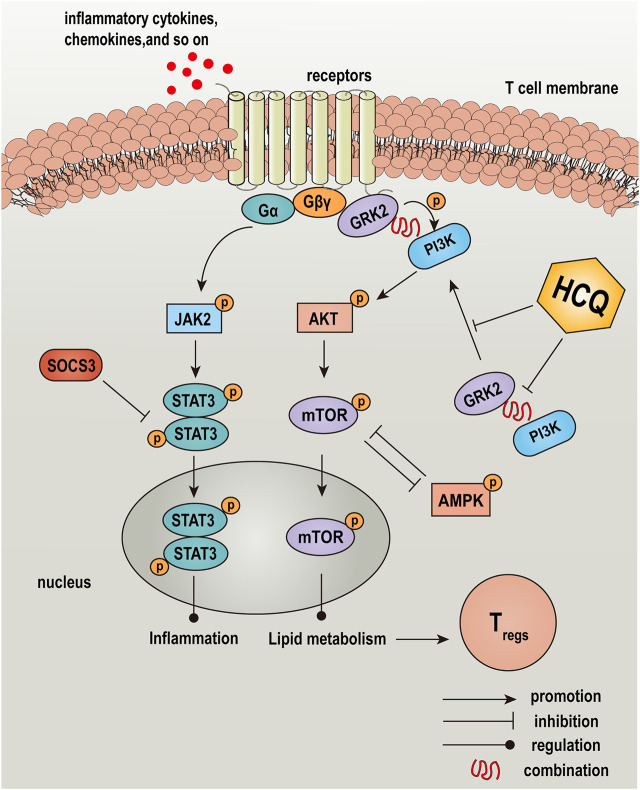
Graphical abstract: a schematic diagram depicted the effect and mechanism of HCQ in the treatment of AIH. HCQ targeted GRK2-PI3K interaction and translocation to modulated lipid metabolism of T lymphocyte, which mediated T cells differentiation and function, and reversed inflammation injury in the liver of AIH.

AMPK is a highly conserved serine/threonine protein kinase, which was the inhibition of mTOR in the lipid oxidation of T_regs_ (Michalek et al., 2011). Our results showed that HCQ significantly increased the expression of p-AMPK compared with ConA pretreatment T lymphocyte, which indicated there was the cross-talk of GRK2/PI3K-AKT and AMPK for HCQ effect in AIH (Figure 9A). We also examined the protein expressions of JAK2-STAT3-SOCS3 pathway in ConA-treated spleen T lymphocytes. The results showed that HCQ suppressed the phosphorylation of JAK2 (Tyr1007), STAT3 (Tyr705), without affecting their total protein expressions, and increased the expressions of SOCS3 (Figure 9B), which appears to indicate the mechanism of anti-inflammatory effect of HCQ. HCQ might have a potential inhibition to signals cross-talk and modulation for immunologic homeostasis in AIH. The multi-targets mechanism of HCQ in the treatment of AIH was shown in Figure 10.

## Discussion

AIH is an autoimmune liver disease, which progress to cirrhosis and liver failure with untreating (Fan et al., 2019). Although the pathogenesis of AIH is not fully understood, it is generally recognized that immunoregulatory networks plays a crucial role (Liberal et al., 2015; Zhu et al., 2021). In a healthy population, circulating autoreactive T cells are suppressed by peripheral tolerance mechanisms to limit autoimmune tissue damage, among which T_reg_-exerted immune suppression plays a key role. Patients with AIH display a reduced T_reg_ frequency or function compared to healthy subjects (Ferri et al., 2010; Sakaguchi et al., 2010; Liberal et al., 2017; Zhu et al., 2021). In addition, several cytokines are implicated in the pathogenesis of ConA-induced hepatitis, of which TNF-α, IFN-γ, TGF-β are the most important (Sass et al., 2002; Wang et al., 2017b; Zhang et al., 2020). HCQ, as an antimalarial agent, has been used for the treatment of immunological diseases: RA and SLE(3). Recently, HCQ exhibited the probable protective effect in liver injury among the COVID-19 patients (Sima, 2021). It was reported that HCQ restored the Th17/T_reg_ balance in MRL/lpr (mouse model that develops SLE) mice, which resulted in a significant decrease in the expression of IL-17 in Th17 cells and a considerable increase in Foxp3 and TGF-β levels (An et al., 2017).

Based on that observation, we established a model of chronic AIH induced by S-100 combined with CFA, which relatively resembled human AIH. Meanwhile, *in vitro*, we tested the effect of HCQ in ConA activated murine spleen T-lymphocyte. The results showed that HCQ ameliorated hepatic pathologic damage and inflammatory infiltration. Besides, HCQ promoted T_reg_ differentiation and the mRNA expression of Foxp3, and retarded CD8^+^T cells differentiation both *in vivo* and *in vitro*. The critical role of HCQ was the inhibition of the metabolism-related GRK2/PI3K-AKT pathway and the inflammation-related JAK2-STAT3 pathway in T cells. The sequence appears to be: ① HCQ acted on GRK2, inhibited the translocation of GRK2, decreased the interaction between GRK2 and PI3K, and reduced the recruitment of PI3K to the membrane ② the blockade of the PI3K-AKT pathway by HCQ accompanied with the inhibition of the inflammation-related JAK2-STAT3 pathway in T cells ③ the diminished lipid metabolism of T cells impaired its function, followed by increasing T_reg_ differentiation, disrupting CD8^+^T cell responses, upregulating co-inhibition signal and anti-inflammatory cytokine TGF-β_1_ expression, and decreasing proinflammatory cytokines released from T cells; and ④ consequently, HCQ inhibited inflammatory cell infiltration and immune responses against liver tissue, and thus diminished autoimmune liver injury (Figure 8C).

Activation of immune cells is associated with a dramatic increase in metabolism. Glucose and lipid metabolism is related to differentiation of T_reg_ (Cluxton et al., 2019; Shan et al., 2020). Besides, it has also been reported that glucose and lipid metabolism is also related to other immune cells, such as CD4^+^T, CD8^+^T, DC, natural killer T (NKT) cells (Palmer et al., 2016; Giovanelli et al., 2019). Our results showed that HCQ treatment decreased NEFA secretion while increased fatty acid oxidation in T lymphocytes, but HCQ had little effect on T cell glucose uptake and glycolysis. Suppressed lipid metabolism predominantly rather than glycolysis of T cell may contribute to immunosuppressive effects of HCQ in AIH. PI3K-AKT-mTOR has been proved to be a classical pathway linking the activation of the insulin receptor to regulate glucose metabolism, which was also shown to be responsible for the accumulation of intracellular lipids by regulating fatty acid synthesis (Chen et al., 2019; Sun et al., 2022). In addition, the activation of PI3K-AKT-mTOR is accompanied with the inhibition of differentiation and function of T_reg_ (Gao et al., 2020). mTOR is the downstream protein of PI3K-AKT, inhibition of mTOR favors naive CD4^+^T-cell conversion to iT_regs_, and mTOR knockout or treatment with the mTOR-specific inhibitor, rapamycin, favors T_reg_ development (Battaglia et al., 2005; Kopf et al., 2007; Delgoffe et al., 2009; Pompura and Dominguez-Villar, 2018). AMPK and mTOR reciprocally regulate each other, which favors FAO and T_reg_ development (Hardie et al., 2006; Hardie, 2011). Our results showed that HCQ may inhibited NEFA secretion, fatty acid synthesis gene expression and promoted FAO through PI3K-AKT-mTOR and AMPK signals. In addition, JAK-STAT is an important pathway mediating most actions of inflammatory cytokines (Xin et al., 2020). HCQ might affect the modulation loop involving interactions of these three signals to maintain immune homeostasis during the progression of AIH. The multi-targets of immune cells and signals affected by HCQ optimized the treatment of HCQ in AIH.

Accumulating data indicate that GRK2 is overexpressed in RA and cardiac dysfunction to play a critical role in ameliorating inflammatory (Penela et al., 2008; Woodall et al., 2016). Previous study proposed that inhibiting the expression of GRK2 in the membrane and increasing its expression in cytoplasm improved the abnormal proliferation of fibroblast like synovial cells in RA(15). In addition, inhibition of GRK2 expression in rat spleen T cells can regulate T cell function to alleviate RA(16). GRK2 specifically recognize and phosphorylate agonist-activated GPCRs, and it also interact with non-GPCRs such as PI3K, AKT (Ribas et al., 2007). The interaction of PI3Kγ with GRK2 mediated PI3K recruitment to the membrane after agonist stimulation. In addition, the inhibition of GRK2-G_βγ_ complex membrane translocation in the fibroblast-like synoviocytes might reduce the membrane expression and activation of PI3K, which mediated the inhibition of AKT activity (Wang et al., 2020). AKT had also been reported to associate with GRK2 directly through the GRK2 C-terminus. GRK2 might inhibit agonist-dependent AKT phosphorylation, although the exact mechanisms were not well established (Ribas et al., 2007). The inhibition of GRK2 rescued AKT activity to promote T_reg_ differentiation in mice with diabetic cardiomyopathy (Han et al., 2020). There was also reported that GRK2 knock down prevented AKT activation in cardiac myocytes but the interaction between GRK2 and AKT was not clear (Penela et al., 2010; Pathania et al., 2019). In addition, increasing PI3K produced polyphosphoinositides that interacted with protein kinases, leading to activation of the kinase AKT (Saltiel, 2021). HCQ has been reported to inhibit PI3K-AKT pathway in renal interstitial fibrosis (Li et al., 2022). Therefore, we tested whether HCQ inhibited the PI3K-AKT pathway by regulating the interaction between GRK2 and PI3K or AKT in T cells of AIH. Our results found that GRK2 and PI3K were co expressed in T lymphocytes. HCQ down-regulated GRK2-PI3K interaction to reduce the complex translocation, and suppressed the phosphorylation of PI3K, AKT, and mTOR. The direct interaction of GRK2 with AKT was undefined. After blocking GRK2, the effect of HCQ was diminished.

In conclusion, HCQ exhibits specific and potent therapeutic effects on AIH and attendant liver injury. The critical role appears to be: HCQ acted on GRK2 translocation mediated PI3K-AKT-mTOR signal inhibition in spleen T lymphocyte, thereby modulating lipid metabolism in T lymphocytes and promoting T_reg_ activity. And HCQ might block the cross-talk of JAK2-STAT3-SOCS3, PI3K-AKT-mTOR and AMPK signals.

## Data Availability

Data supporting the results of this study can be obtained from the corresponding author upon reasonable request.

## References

[B1] An N. Chen Y. Wang C. Yang C. Wu Z. H. Xue J. (2017). Chloroquine autophagic inhibition rebalances Th17/treg-mediated immunity and ameliorates systemic lupus erythematosus. Cell. Physiol. biochem. 44 (1), 412–422. 10.1159/000484955 29141242

[B2] Battaglia M. Stabilini A. Roncarolo M. G. (2005). Rapamycin selectively expands CD4+CD25+FoxP3+ regulatory T cells. Blood 105 (12), 4743–4748. 10.1182/blood-2004-10-3932 15746082

[B3] Beck T. C. Beck K. R. Holloway C. B. Hemings R. A. Jr. Dix T. A. Norris R. A. (2020). The C-C chemokine receptor type 4 is an immunomodulatory target of hydroxychloroquine. Front. Pharmacol. 11, 1253. 10.3389/fphar.2020.01253 32973504 PMC7482581

[B4] Ben-Zvi I. Kivity S. Langevitz P. Shoenfeld Y. (2012). Hydroxychloroquine: From malaria to autoimmunity. Clin. Rev. Allergy Immunol. 42 (2), 145–153. 10.1007/s12016-010-8243-x 21221847 PMC7091063

[B5] Chen Q. Tang L. Xin G. Li S. Ma L. Xu Y. (2019). Oxidative stress mediated by lipid metabolism contributes to high glucose-induced senescence in retinal pigment epithelium. Free Radic. Biol. Med. 130, 48–58. 10.1016/j.freeradbiomed.2018.10.419 30339883

[B6] Cheng J. Lucas P. C. McAllister-Lucas L. M. (2021). Canonical and non-canonical roles of GRK2 in lymphocytes. Cells 10 (2), 307. 10.3390/cells10020307 33546162 PMC7913175

[B7] Christen U. (2019). Animal models of autoimmune hepatitis. Biochim. Biophys. Acta. Mol. Basis Dis. 1865 (5), 970–981. 10.1016/j.bbadis.2018.05.017 29857050

[B8] Cluxton D. Petrasca A. Moran B. Fletcher J. M. (2019). Differential regulation of human Treg and Th17 cells by fatty acid synthesis and glycolysis. Front. Immunol. 10, 115. 10.3389/fimmu.2019.00115 30778354 PMC6369198

[B9] Delgoffe G. M. Kole T. P. Zheng Y. Zarek P. E. Matthews K. L. Xiao B. (2009). The mTOR kinase differentially regulates effector and regulatory T cell lineage commitment. Immunity 30 (6), 832–844. 10.1016/j.immuni.2009.04.014 19538929 PMC2768135

[B10] Fan X. Men R. Wang H. Shen M. Wang T. Ye T. (2019). Methylprednisolone decreases mitochondria-mediated apoptosis and autophagy dysfunction in hepatocytes of experimental autoimmune hepatitis model via the akt/mTOR signaling. Front. Pharmacol. 10, 1189. 10.3389/fphar.2019.01189 31680966 PMC6813226

[B11] Fang X. Tan T. Gao B. Zhao Y. Liu T. Xia Q. (2020). Germacrone regulates HBXIP-mediated cell cycle, apoptosis and promotes the formation of autophagosomes to inhibit the proliferation of gastric cancer cells. Front. Oncol. 10, 537322. 10.3389/fonc.2020.537322 33244453 PMC7683780

[B12] Ferri S. Longhi M. S. De Molo C. Lalanne C. Muratori P. Granito A. (2010). A multifaceted imbalance of T cells with regulatory function characterizes type 1 autoimmune hepatitis. Hepatology 52 (3), 999–1007. 10.1002/hep.23792 20683931

[B13] Fox C. J. Hammerman P. S. Thompson C. B. (2005). Fuel feeds function: Energy metabolism and the T-cell response. Nat. Rev. Immunol. 5 (11), 844–852. 10.1038/nri1710 16239903

[B14] Gao L. Dong Y. Lin R. Meng Y. Wu F. Jia L. (2020). The imbalance of Treg/Th17 cells induced by perinatal bisphenol A exposure is associated with activation of the PI3K/Akt/mTOR signaling pathway in male offspring mice. Food Chem. Toxicol. 137, 111177. 10.1016/j.fct.2020.111177 32028014

[B15] Giovanelli P. Sandoval T. A. Cubillos-Ruiz J. R. (2019). Dendritic cell metabolism and function in tumors. Trends Immunol. 40 (8), 699–718. 10.1016/j.it.2019.06.004 31301952

[B16] Han C. C. Ma Y. Li Y. Wang Y. Wei W. (2016). Regulatory effects of GRK2 on GPCRs and non-GPCRs and possible use as a drug target (Review). Int. J. Mol. Med. 38 (4), 987–994. 10.3892/ijmm.2016.2720 27573285

[B17] Han Y. Lai J. Tao J. Tai Y. Zhou W. Guo P. (2020). Sustaining circulating regulatory T cell subset contributes to the therapeutic effect of paroxetine on mice with diabetic cardiomyopathy. Circ. J. 84 (9), 1587–1598. 10.1253/circj.CJ-19-1182 32741881

[B18] Hardie D. G. (2011). AMP-Activated protein kinase: An energy sensor that regulates all aspects of cell function. Genes. Dev. 25 (18), 1895–1908. 10.1101/gad.17420111 21937710 PMC3185962

[B19] Hardie D. G. Hawley S. A. Scott J. W. (2006). AMP-activated protein kinase--development of the energy sensor concept. J. Physiol. 574 (1), 7–15. 10.1113/jphysiol.2006.108944 16644800 PMC1817788

[B20] Hu C. Lu L. Wan J. P. Wen C. (2017). The pharmacological mechanisms and therapeutic activities of hydroxychloroquine in rheumatic and related diseases. Curr. Med. Chem. 24 (20), 2241–2249. 10.2174/0929867324666170316115938 28302011

[B21] Kopf H. de la Rosa G. M. Howard O. M. Chen X. (2007). Rapamycin inhibits differentiation of Th17 cells and promotes generation of FoxP3+ T regulatory cells. Int. Immunopharmacol. 7 (13), 1819–1824. 10.1016/j.intimp.2007.08.027 17996694 PMC2223142

[B22] Koyama Y. Brenner D. A. (2017). Liver inflammation and fibrosis. J. Clin. Invest. 127 (1), 55–64. 10.1172/JCI88881 28045404 PMC5199698

[B23] Li D. Yu K. Feng F. Zhang Y. Bai F. Zhang Y. (2022). Hydroxychloroquine alleviates renal interstitial fibrosis by inhibiting the PI3K/Akt signaling pathway. Biochem. Biophys. Res. Commun. 610, 154–161. 10.1016/j.bbrc.2022.04.058 35462097

[B24] Liberal R. Grant C. R. Longhi M. S. Mieli-Vergani G. Vergani D. (2015). Regulatory T cells: Mechanisms of suppression and impairment in autoimmune liver disease. IUBMB Life 67 (2), 88–97. 10.1002/iub.1349 25850692

[B25] Liberal R. Grant C. R. Yuksel M. Graham J. Kalbasi A. Ma Y. (2017). Regulatory T-cell conditioning endows activated effector T cells with suppressor function in autoimmune hepatitis/autoimmune sclerosing cholangitis. Hepatology 66 (5), 1570–1584. 10.1002/hep.29307 28597951 PMC5689077

[B26] Liu T. Cao H. Ji Y. Pei Y. Yu Z. Quan Y. (2015). Interaction of dendritic cells and T lymphocytes for the therapeutic effect of Dangguiliuhuang decoction to autoimmune diabetes. Sci. Rep. 5, 13982. 10.1038/srep13982 26358493 PMC4566122

[B27] Liu T. T. Ding T. L. Ma Y. Wei W. (2014). Selective α1B- and α1D-adrenoceptor antagonists suppress noradrenaline-induced activation, proliferation and ECM secretion of rat hepatic stellate cells *in vitro* . Acta Pharmacol. Sin. 35 (11), 1385–1392. 10.1038/aps.2014.84 25283507 PMC4220077

[B28] Lyu X. Zeng L. Zhang H. Ke Y. Liu X. Zhao N. (2020). Hydroxychloroquine suppresses lung tumorigenesis via inducing FoxO3a nuclear translocation through STAT3 inactivation. Life Sci. 246, 117366. 10.1016/j.lfs.2020.117366 32001266

[B29] Martinez G. P. Zabaleta M. E. Di Giulio C. Charris J. E. Mijares M. R. (2020). The role of chloroquine and hydroxychloroquine in immune regulation and diseases. Curr. Pharm. Des. 26 (35), 4467–4485. 10.2174/1381612826666200707132920 32634079

[B30] Matias M. I. Yong C. S. Foroushani A. Goldsmith C. Mongellaz C. Sezgin E. (2021). Regulatory T cell differentiation is controlled by αKG-induced alterations in mitochondrial metabolism and lipid homeostasis. Cell Rep. 37 (5), 109911. 10.1016/j.celrep.2021.109911 34731632 PMC10167917

[B31] Michalek R. D. Gerriets V. A. Jacobs S. R. Macintyre A. N. MacIver N. J. Mason E. F. (2011). Cutting edge: Distinct glycolytic and lipid oxidative metabolic programs are essential for effector and regulatory CD4+ T cell subsets. J. Immunol. 186 (6), 3299–3303. 10.4049/jimmunol.1003613 21317389 PMC3198034

[B32] Murphy K. M. Heimberger A. B. Loh D. Y. (1990). Induction by antigen of intrathymic apoptosis of CD4+CD8+TCRlo thymocytes *in vivo* . Science 250 (4988), 1720–1723. 10.1126/science.2125367 2125367

[B33] Palmer C. S. Hussain T. Duette G. Weller T. J. Ostrowski M. Sada-Ovalle I. (2016). Regulators of glucose metabolism in CD4(+) and CD8(+) T cells. Int. Rev. Immunol. 35 (6), 477–488. 10.3109/08830185.2015.1082178 26606199

[B34] Pathania A. S. Ren X. Mahdi M. Y. Shackleford G. M. Erdreich-Epstein A. (2019). GRK2 promotes growth of medulloblastoma cells and protects them from chemotherapy-induced apoptosis. Sci. Rep. 9 (1), 13902. 10.1038/s41598-019-50157-5 31554835 PMC6761358

[B35] Penela P. Murga C. Ribas C. Lafarga V. Mayor F. Jr (2010). The complex G protein-coupled receptor kinase 2 (GRK2) interactome unveils new physiopathological targets. Br. J. Pharmacol. 160 (4), 821–832. 10.1111/j.1476-5381.2010.00727.x 20590581 PMC2935989

[B36] Penela P. Murga C. Ribas C. Salcedo A. Jurado-Pueyo M. Rivas V. (2008). G protein-coupled receptor kinase 2 (GRK2) in migration and inflammation. Arch. Physiol. Biochem. 114 (3), 195–200. 10.1080/13813450802181039 18618354

[B37] Pompura S. L. Dominguez-Villar M. (2018). The PI3K/AKT signaling pathway in regulatory T-cell development, stability, and function. J. Leukoc. Biol. 103, 1065–1076. 10.1002/JLB.2MIR0817-349R 29357116

[B38] Rainsford K. D. Parke A. L. Clifford-Rashotte M. Kean W. F. (2015). Therapy and pharmacological properties of hydroxychloroquine and chloroquine in treatment of systemic lupus erythematosus, rheumatoid arthritis and related diseases. Inflammopharmacology 23 (5), 231–269. 10.1007/s10787-015-0239-y 26246395

[B39] Ribas C. Penela P. Murga C. Salcedo A. Garcia-Hoz C. Jurado-Pueyo M. (2007). The G protein-coupled receptor kinase (GRK) interactome: Role of GRKs in GPCR regulation and signaling. Biochim. Biophys. Acta 1768 (4), 913–922. 10.1016/j.bbamem.2006.09.019 17084806

[B40] Sakaguchi S. Miyara M. Costantino C. M. Hafler D. A. (2010). FOXP3+ regulatory T cells in the human immune system. Nat. Rev. Immunol. 10 (7), 490–500. 10.1038/nri2785 20559327

[B41] Saltiel A. R. (2021). Insulin signaling in health and disease. J. Clin. Invest. 131 (1), 142241. 10.1172/JCI142241 33393497 PMC7773347

[B42] Sass G. Heinlein S. Agli A. Bang R. Schumann J. Tiegs G. (2002). Cytokine expression in three mouse models of experimental hepatitis. Cytokine 19 (3), 115–120. 10.1006/cyto.2002.1948 12242077

[B43] Seenappa V. Das B. Joshi M. B. Satyamoorthy K. (2016). Context dependent regulation of human phosphoenolpyruvate carboxykinase isoforms by DNA promoter methylation and RNA stability. J. Cell. Biochem. 117 (11), 2506–2520. 10.1002/jcb.25543 26990534

[B44] Shan J. Jin H. Xu Y. (2020). T cell metabolism: A new perspective on Th17/treg cell imbalance in systemic lupus erythematosus. Front. Immunol. 11, 1027. 10.3389/fimmu.2020.01027 32528480 PMC7257669

[B45] Sima A. R. (2021). Hydroxychloroquine's probable protective effect in liver injury among the COVID-19 patients. Middle East J. Dig. Dis. 13 (1), 80–81. 10.34172/mejdd.2021.209 34712444 PMC8531938

[B46] Sun J. P. Shi L. Wang F. Qin J. Ke B. (2022). Modified linggui zhugan decoction ameliorates glycolipid metabolism and inflammation via PI3K-Akt/mTOR-S6K1/AMPK-PGC-1 alpha signaling pathways in obese type 2 diabetic rats. Chin. J. Integr. Med. 28 (1), 52–59. 10.1007/s11655-020-3285-2 33211278

[B47] Wang D. D. Jiang M. Y. Wang W. Zhou W. J. Zhang Y. W. Yang M. (2020). Paeoniflorin-6'-O-benzene sulfonate down-regulates CXCR4-Gβγ-PI3K/AKT mediated migration in fibroblast-like synoviocytes of rheumatoid arthritis by inhibiting GRK2 translocation. Biochem. Biophys. Res. Commun. 526 (3), 805–812. 10.1016/j.bbrc.2020.03.164 32268958

[B48] Wang L. Zhang W. Ge C. H. Yin R. H. Xiao Y. Zhan Y. Q. (2017). Toll-like receptor 5 signaling restrains T-cell/natural killer T-cell activation and protects against concanavalin A-induced hepatic injury. Hepatology 65 (6), 2059–2073. 10.1002/hep.29140 28273362

[B49] Wang Q. Wang L. Wu L. Zhang M. Hu S. Wang R. (2017). Paroxetine alleviates T lymphocyte activation and infiltration to joints of collagen-induced arthritis. Sci. Rep. 7, 45364. 10.1038/srep45364 28349925 PMC5368980

[B50] Wang Y. Han C. C. Cui D. Luo T. T. Li Y. Zhang Y. (2018). Immunomodulatory effects of CP-25 on splenic T cells of rats with adjuvant arthritis. Inflammation 41 (3), 1049–1063. 10.1007/s10753-018-0757-z 29473135

[B51] Woodall M. C. Woodall B. P. Gao E. Yuan A. Koch W. J. (2016). Cardiac fibroblast GRK2 deletion enhances contractility and remodeling following ischemia/reperfusion injury. Circ. Res. 119 (10), 1116–1127. 10.1161/CIRCRESAHA.116.309538 27601479 PMC5085864

[B52] Xiang M. Liu T. Tan W. Ren H. Li H. Liu J. (2016). Effects of kinsenoside, a potential immunosuppressive drug for autoimmune hepatitis, on dendritic cells/CD8(+) T cells communication in mice. Hepatology 64 (6), 2135–2150. 10.1002/hep.28825 27639182

[B53] Xiang M. Liu T. Tian C. Ma K. Gou J. Huang R. (2022). Kinsenoside attenuates liver fibro-inflammation by suppressing dendritic cells via the PI3K-AKT-FoxO1 pathway. Pharmacol. Res. 177, 106092. 10.1016/j.phrs.2022.106092 35066108 PMC8776354

[B54] Xie B. Geng Q. Xu J. Lu H. Luo H. Hu Y. (2020). The multi-targets mechanism of hydroxychloroquine in the treatment of systemic lupus erythematosus based on network pharmacology. Lupus 29 (13), 1704–1711. 10.1177/0961203320952541 32854577

[B55] Xie B. Lu H. Xu J. Luo H. Hu Y. Chen Y. (2021). Targets of hydroxychloroquine in the treatment of rheumatoid arthritis. A network pharmacology study. Jt. Bone Spine 88 (2), 105099. 10.1016/j.jbspin.2020.105099 33160044

[B56] Xin P. Xu X. Deng C. Liu S. Wang Y. Zhou X. (2020). The role of JAK/STAT signaling pathway and its inhibitors in diseases. Int. Immunopharmacol. 80, 106210. 10.1016/j.intimp.2020.106210 31972425

[B57] Yang H. Cao Q. Xiong X. Zhao P. Shen D. Zhang Y. (2020). Fluoxetine regulates glucose and lipid metabolism via the PI3KAKT signaling pathway in diabetic rats. Mol. Med. Rep. 22 (4), 3073–3080. 10.3892/mmr.2020.11416 32945450 PMC7453494

[B58] Yang Y. Zhang P. Wang Y. Wei S. Zhang L. Wang J. (2018). Hepatoprotective effect of san-cao granule on con A-induced liver injury in mice and mechanisms of action exploration. Front. Pharmacol. 9, 624. 10.3389/fphar.2018.00624 29946260 PMC6005824

[B59] Zhang M. Li Q. Zhou C. Zhao Y. Li R. Zhang Y. (2020). Demethyleneberberine attenuates concanavalin A-induced autoimmune hepatitis in mice through inhibition of NF-κB and MAPK signaling. Int. Immunopharmacol. 80, 106137. 10.1016/j.intimp.2019.106137 31931366

[B60] Zheng H. Zhang Y. He J. Yang Z. Zhang R. Li L. (2021). Hydroxychloroquine inhibits macrophage activation and attenuates renal fibrosis after ischemia-reperfusion injury. Front. Immunol. 12, 645100. 10.3389/fimmu.2021.645100 33936063 PMC8079743

[B61] Zhu H. Liu Z. An J. Zhang M. Qiu Y. Zou M. H. (2021). Activation of AMPKα1 is essential for regulatory T cell function and autoimmune liver disease prevention. Cell. Mol. Immunol. 18 (12), 2609–2617. 10.1038/s41423-021-00790-w 34728795 PMC8632917

