# Corrigendum: Hydroxychloroquine attenuates autoimmune hepatitis by suppressing the interaction of GRK2 with PI3K in T lymphocytes

**DOI:** 10.3389/fphar.2023.1322549

**Published:** 2023-11-13

**Authors:** Chao Jin, Bei-Bei Gao, Wen-Jing Zhou, Bao-Jing Zhao, Xing Fang, Chun-Lan Yang, Xiao-Hua Wang, Quan Xia, Ting-Ting Liu

**Affiliations:** ^1^ School of Pharmacy, The First Affiliated Hospital of Anhui Medical University, Anhui Medical University, Hefei, China; ^2^ The Grade 3 Pharmaceutical Chemistry Laboratory of State Administration of Traditional Chinese Medicine, Hefei, China; ^3^ Department of Pharmacy, The Second Hospital of Anhui Medical University, Hefei, China; ^4^ Department of Obstetrics and Gynecology, The First Affiliated Hospital of Anhui Medical University, Hefei, China; ^5^ Department of Pharmacy, The Second People’s Hospital of Hefei, Hefei Hospital Affiliated to Anhui Medical University, Hefei, China

**Keywords:** hydroxychloroquine, autoimmune hepatitis, regulatory T cells, glycolipid metabolism, G protein-coupled receptor kinase 2, PI3K-AKT axis

In the published article, there was an error in affiliations **1, 2**. Instead of “^1^School of Pharmacy, Anhui Medical University, Hefei, China, ^2^Department of Pharmacy, The First Affiliated Hospital of Anhui Medical University, The Grade 3 Pharmaceutical Chemistry Laboratory of State Administration of Traditional Chinese Medicine, Hefei, China,” it should be “^1^School of Pharmacy, the First Affiliated Hospital of Anhui Medical University, Anhui Medical University, Hefei, China, ^2^The Grade 3 Pharmaceutical Chemistry Laboratory of State Administration of Traditional Chinese Medicine, Hefei, China”.

The authors apologize for this error and state that this does not change the scientific conclusions of the article in any way. The original article has been updated.

