# Supplementary material for: Hydroxychloroquine attenuates autoimmune hepatitis by suppressing the interaction of GRK2 with PI3K in T lymphocytes
Source: Front Pharmacol. 2022 Sep 15;13:972397. doi: 10.3389/fphar.2022.972397 (PMC9520598; doi:10.3389/fphar.2022.972397)
Supplement: Supplementary file 1 [file Table1.DOCX]

**Supplementary Table 1. Antibody information for Western blot**

| **Antibody** | **Manufacturer** | **Catalog number** | **Dilution used** |
| --- | --- | --- | --- |
| β-actin | Proteintech | 66009-1-lg | 1:10000 |
| GRK2 | Santa | Sc-13143 | 1:500 |
| PI3K | Immunoway | YM3800 | 1:1000 |
| p-PI3K(Tyr467/199) | Immunoway | YP0224 | 1:1000 |
| AKT | Proteintech | 10176-2-AP | 1:2000 |
| p-AKT(Ser473) | Proteintech | 66444-1-lg | 1:2000 |
| mTOR | Proteintech | 20657-1-AP | 1:400 |
| p-mTOR (Ser2448) | Immunoway | YP0176 | 1:1000 |
| JAK2 | Immunoway | YT2429 | 1:1000 |
| p-JAK2(Tyr1007) | Immunoway | YP0155 | 1:1000 |
| STAT3 | Proteintech | 10253-2-AP | 1:1000 |
| p-STAT3（Tyr705） | Immunoway | YP0251 | 1:1000 |
| SOCS3 | Proteintech | 14025-1-AP | 1:1000 |
| AMPKα | Cell Signaling | 5832T | 1:1000 |
| P-AMPKα(Thr172) | Cell Signaling | 2535T | 1:1000 |
| ATP1A1 | Zenbio | 380790 | 1:1000 |

**Supplementary Table 2. The sequences of primers**.

| **Gene name** | **Forward sequence** | **Reverse sequence** |
| --- | --- | --- |
| *β-actin* | ACACCTTCTACAATGAGCTG | CTGCTTGCTGATCCACATCT |
| *AldoA* | CGTGTGAATCCCTGCATTGG | CAGCCCCTGGGTAGTTGTC |
| *AldoB* | GAAACCGCCTGCAAAGGATAA | GAGGGTCTCGTGGAAAAGGAT |
| *AldoC* | AGAAGGAGTTGTCGGATATTGCT | TTCTCCACCCCAATTTGGCTC |
| *Enolase1* | TGCGTCCACTGGCATCTAC | CAGAGCAGGCGCAATAGTTTTA |
| *Enolase2* | GTCCCTGGCCGTGTGTAAG | CATCCCGAAAGCTCTCAGC |
| *Enolase3* | CACAGCCAAGGGTCGATTCC | CCCAGGTATCGTGCTTTGTCT |
| *Glut1* | GAGTGTGGTGGATGGGATG | AACACTGGTGTCATCAACGC |
| *Gpi* | TCAAGCTGCGCGAACTTTTTG | GGTTCTTGGAGTAGTCCACCAG |
| *HK2* | TGATCGCCTGCTTATTCACGG | AACCGCCTAGAAATCTCCAGA |
| *HK4* | TGAGCCGGATGCAGAAGGA | GCAACATCTTTACACTGGCCT |
| *MCT4* | TCACGGGTTTCTCCTACGC | GCCAAAGCGGTTCACACAC |
| *Pfkl* | GGAGGCGAGAACATCAAGCC | CGGCCTTCCCTCGTAGTGA |
| *Pfkfb1* | ATGAGCTGCCCTATCTCAAGT | GTCCCGGTGTGTGTTCACAG |
| *PKM* | GCCGCCTGGACATTGACTC | CCATGAGAGAAATTCAGCCGAG |
| *Foxp3* | GACATCCCATATTCTCCCA | GACGTGAAGCCTAGACAGC |
| *PD-1* | CGCCTGAGTCCCAGCAACCAG | GCTCCAGGGCTCTCCTCGAT |
| *CTLA-4*  *CD28*  *GzmB*  *GRK2*  *PI3K*  *AKT*  *mTOR*  *SREBP1*  *SREBP2*  *PPARα*  *PPARγ*  *FAS*  *FASN*  *Acaca*  *Elovl6*  *CD36*  *Lss*  *Hmgcs*  *Pmvk*  *SirT1*  *SirT2*  *SirT3* | TGGGCTTCCTAGATTACCCCTT  ACCTATCAGCCCCAGTTTCGC  GACCCTACATGGCCTTACTTTC  GGGGACGTGTTCCAGAAGTTC  TGCTGGACACCTGTTGGG  CCCTGGATCTTATGTGCC  TCCTTACGGTTTCCTTCTCC  TGACCCGGCTATTCCGTGA  TGGGCGATGAGCTGACTCT  GGGTACCACTACGGAGTTCACG  GTGCCAGTTTCGATCCGTAGA  GCACAGCAACCAGCAATACA  GGCTCTATGGATTACCCAAGC  CTCCCGATTCATAATTGGGTCTG  GAAAAGCAGTTCAACGAGAACG  TTGGCCAAGCTATTGCGACA  GGACTTACCAAAAGCGCAAAC  CGGATCGTGAAGACATCAACTC  AAAATCCGGGAAGGACTTCGT  GATGACGATGACAGAACGTCACA  CTCATCAGCAAGGCACCACTAG  ACAGCTACATGCACGGTCTG | AATCTGCGTCCCGTTGCCCAT  TTCCATTGCTCCTCTCGTTGT  TATGAAGCCAGTCTTTGCAGTC  ATTCGATGCACACTGAAGTCAT  TCGCTCCTTCTTGTAGTCGTAC  TCTTGAGGAGGAAGTAGCG  TCAGGTCCACTCCATCCC  CTGGGCTGAGCAATACAGTTC  CAAATCAGGGAACTCTCCCAC  CAGACAGGCACTTGTGAAAACG  GGCCAGCATCGTGTAGATGA  TCAGCAATTCTCGGGATGTA  CCAGTGTTCGTTCCTCGGA  TCGACCTTGTTTTACTAGGTGC  AGATGCCGACCACCAAAGATA  GCAAAGGCATTGGCTGGAAG  GAAGAGCGGACCACCATAATC  CGCCCAATGCAATCATAGGAA  AGAGCACAGATGTTACCTCCA  GGATCGGTGCCAATCATGAG  CCATCATCATGCCCAGGAA  TGCTCCCCAAAGAACACAAT |
